# Adjustment of familial relatedness in association test for rare variants

**DOI:** 10.1186/1753-6561-8-S1-S39

**Published:** 2014-06-17

**Authors:** Cong Li, Can Yang, Mengjie Chen, Xiaowei Chen, Lin Hou, Hongyu Zhao

**Affiliations:** 1 Program in Computational Biology and Bioinformatics, Yale University, New Haven, CT 06520, USA; 2 Department of Biostatistics, Yale School of Public Health, 60 College Street, New Haven, CT 06520, USA

## Abstract

High-throughput sequencing technology allows researchers to test associations between phenotypes and all the variants identified throughout the genome, and is especially useful for analyzing rare variants. However, the statistical power to identify phenotype-associated rare variants is very low with typical genome-wide association studies because of their low allele frequencies among unrelated individuals. In contrast, a family-based design may have more power because rare variants are more likely to be enriched in families than among unrelated individuals. Regardless, an analysis of family-based association studies needs to account appropriately for relatedness between family members. We analyzed the observed quantitative trait systolic blood pressure as well as the simulated Q1 data in the Genetic Analysis Workshop 18 data set using 4 tests: (a) a single-variant test, (b) a collapsing test, (c) a single-variant test where familial relatedness was accounted for, and (d) a collapsing test where familial relatedness was accounted for. We then compared the results of the 4 methods and observed that adjusting for familial relatedness could appropriately control the false-positive rate while maintaining reasonable power to detect several strongly associated variants/genes.

## Background

Current platforms for genome-wide association studies are limited to scanning common variants. Although rare variants may contribute a significant proportion of heritability, the statistical power to detect rare variants is low because of their low allele frequencies. Recent advances in high-throughput sequencing technologies have provided us great opportunities to delve deeper into the genetic components of complex traits by identifying millions of rare variants in the human genome [1] and allowing them to be tested for associations with complex traits.

In an effort to increase the power to detect rare-variant associations, many methods have been proposed to aggregate the effects of multiple rare variants within a specific functional unit, for example, a gene [2]. Among those methods, the kernel score test has enjoyed great popularity thanks to its flexibility and computational efficiency [3]. Family-based designs may offer more power, however, because related individuals are more likely than unrelated individuals to be enriched for rare variants [4]. One challenge of analyzing family data is that familial relatedness needs to be carefully adjusted for in the association analysis. Linear mixed models have been used to account for population stratification in the context of genome-wide association studies (GWAS) [5], and can also be naturally adapted to a family-based design. In this paper, we adopted a linear mixed model to adjust for familial relatedness while aggregating the effects of rare variants within a gene using a kernel score test.

## Methods

### Data description and preprocessing

The Genetic Analysis Workshop 18 (GAW18) data consists of genotyping and sequencing data for 959 individuals from 20 extended families. Among them, 849 individuals had at least 1 blood pressure measurement. For the real data, we used the first nonmissing measurement of systolic blood pressure (SBP) as the target quantitative trait. For the 200 replicates of the simulated phenotype, we also used the first measurement of SBP as the target quantitative trait. Finally, to evaluate the false-positive rate, we used the simulated Q1 data as the null phenotype.

The common variants in the genotyping data were used to estimate the genetic similarity matrix. As for the sequencing data, we selected only the nonsynonymous mutations for the association test in recognition of the fact that variants causing amino acid alterations tend to have large effects on the phenotype.

### The model

We used the following linear mixed model to adjust for familial relatedness: Y=Cγ+Xβ+Zα+e where **Y **represents the phenotype (quantitative trait), **C **represents the collection of the covariates (age and gender), **X **includes the genotypes of the variants to be tested, and **Z **is the design matrix for the whole-genome polygenic random effects *α*. Finally, *γ *and *β *are fixed effects and **e **is the random residual. When the random effects are integrated out, the model is marginalized as $\text{Y}\sim \text{N}\left(\text{C}\gamma +\text{X}\beta ,\text{K}\sigma_{\alpha }^{2}+\text{I}\sigma_{e}^{2}\right),$ where $\sigma_{\alpha }^{2}$ and $\sigma_{e}^{2}$ are the variances for the random effects *α *and e, respectively. As for **K**, one could use twice the theoretical kinship matrix to represent the familial relationships. However, to account for potential cryptic relatedness between individuals across different families, we used a genetic similarity matrix that was estimated from the genotyping data as follows: ${\text{K}}_{ij}=\frac{1}{p}\overset{p}{\underset{m=1}{\sum }}\frac{\left(z_{im}-2f_{m}\right)\left(z_{jm}-2f_{m}\right)}{2f_{m}\left(1-f_{m}\right)},$ where $z_{im}$ is the number of minor alleles for *i*th individual at the *m*th marker, $f_{m}$ is the allele frequency for the *m*th marker, and *p *is the total number of markers in the genotyping data.

We investigated 4 methods of association analysis: (a) a single-variant association test without adjusting for familial relatedness (UNI), that is, the component Zα is excluded in the model; (b) a single-variant association test adjusting for familial relatedness (UNI-ADJ); (c) a kernel score test without adjusting for familial relatedness (SKAT); and (d) a kernel score test adjusting for familial relatedness (SKAT-ADJ). The first method is a simple linear regression. The second method tests the null hypothesis of zero fixed effects in a linear mixed model. Specifically, we used the likelihood ratio test implemented in the software GEMMA [6]. The third method is the direct application of the SKAT software [3] that ignores familial relatedness. As for the fourth method, we adopted the approach described in Ref. [7]. Specifically, we estimated the covariance matrix $\text{R}=\text{cov}\;\left(\text{Z}\alpha +\text{e}\right)=\text{K}\sigma_{\alpha }^{2}+\text{I}\sigma_{e}^{2}$ under the null model without genes or variants being tested. Then we applied a data transformation to calculate Ỹ,$\tilde{\text{X}},$ and $\tilde{\text{C}},$ where: $\text{Ỹ}={\text{R}}^{-1/2}\text{Y},$$\tilde{\text{X}}={\text{R}}^{-1/2}\text{X},$$\tilde{\text{C}}={\text{R}}^{-1/2}\text{C},$ leading to the following transformed model, $\text{Ỹ}=\tilde{\text{C}}\gamma +\tilde{\text{X}}\beta +\text{ẽ},$ where $\text{ẽ}={\text{R}}^{-1/2}\left(\text{Z}\alpha +\text{e}\right)$. Note that each ${\text{ẽ}}_{i}$ is independent and identically distributed; therefore, the kernel score test can be appropriately applied on the transformed data.

## Results

### Simulated data

For the simulated data, we analyzed the genes that explain the highest percentages of SBP variance (the percentages of SBP variance explained by each gene in the simulation were provided by the GAW18 organizers) using the 4 methods described above. Genes without nonsynonymous mutations were excluded. For the first two single-variant methods, we performed a Bonferroni correction on the smallest *p *value of the variants in a gene to obtain the gene-level *p *value. To assess the false-positive rate, we also calculated the *p *values using Q1 as the phenotype. The power and the false-positive rate were calculated based on the 200 replicates. Table 1 shows the results for the top 7 genes. As the table shows, ignoring familial relatedness results in inflation of the false-positive rate using the UNI and SKAT methods, in contrast with the UNI-ADJ and SKAT-ADJ methods, which still demonstrate good control of the false-positive rate. As for the power, the single-variant method does better for genes whose effects on the phenotype are dominated by a single variant (eg, *LEPR*) whereas the collapsing method is more powerful for genes containing multiple variants with comparable effect sizes.

**Table 1:** The power and false-positive rate (FPR) of the 4 methods in simulated data

Gene		UNI		UNI-ADJ		SKAT		SKAT-ADJ	
		
	# of NS variants	Power	FPR	Power	FPR	Power	FPR	Power	FPR
*MAP4*	8	1	0.12	1	0.04	1	0.13	1	0.03

*TNN*	20	0.9	0.225	0.74	0.055	0.895	0.38	0.825	0.04

*LEPR*	6	0.915	0.125	0.895	0.05	0.22	0.085	0.73	0.04

*FLT3*	4	0.66	0.105	0.3	0.045	0.33	0.135	0.305	0.07

*FLNB*	13	0.44	0.14	0.165	0.03	0.08	0.17	0.515	0.035

*ZNF443*	9	0.070	0.155	0.035	0.030	0.150	0.075	0.050	0.075

*GSN*	6	0.265	0.155	0.130	0.070	0.030	0.065	0.385	0.065

A significance level of 0.05 was used to calculate both power and FPR.

### Real data

**Figure 1:**
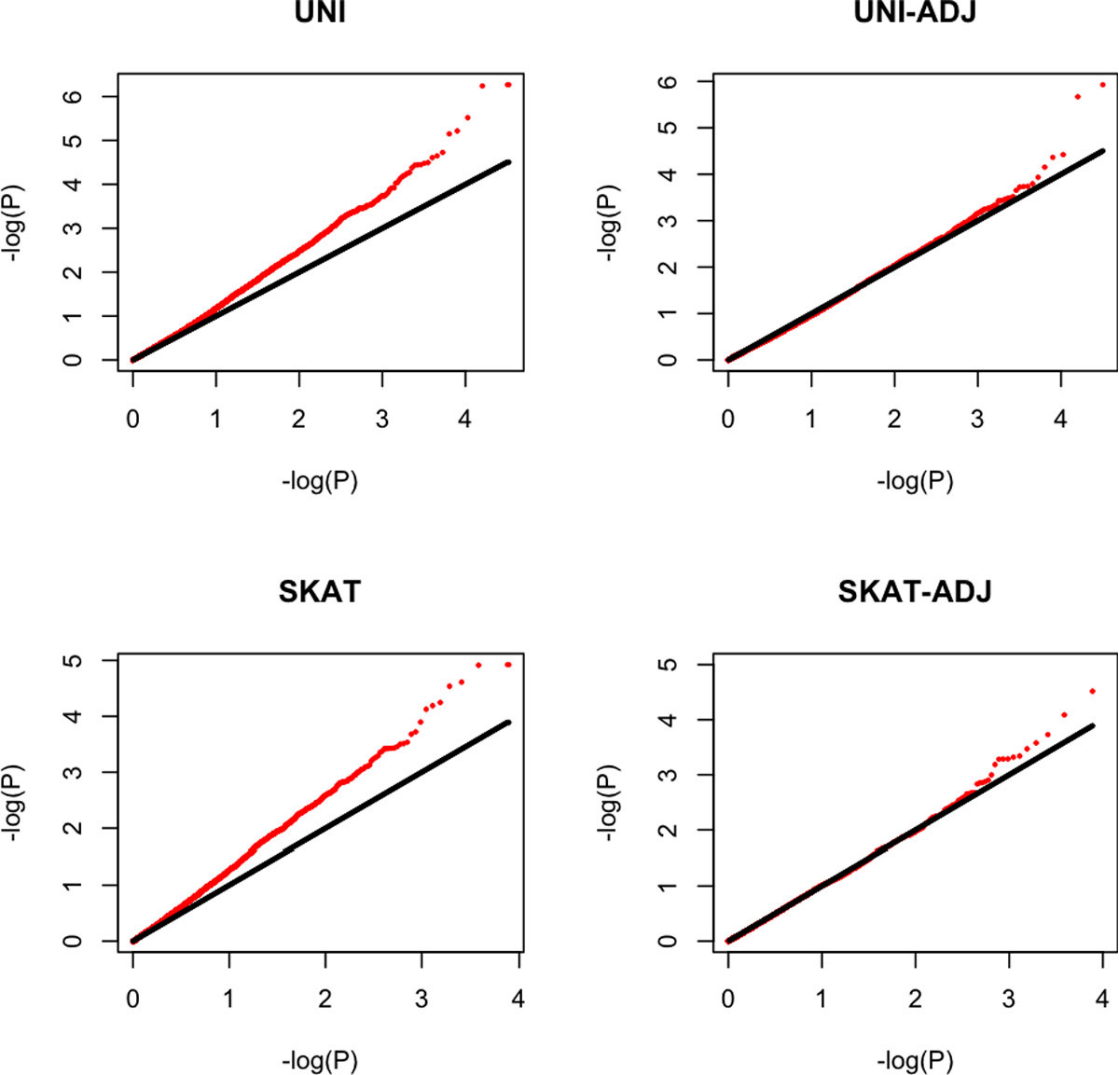
**Q-Q plots for the 4 methods in real data**. The Q-Q plots are based on −log10 *p*-values of the 7781 genes with at least 1 nonsynonymous mutation in the real data. Red curves represent the observed *p *values and the black curves represent the expected *p *values under the null model.

Consistent with our observation in the simulated data, the Q-Q plot (Figure 1) shows that the UNI and SKAT methods that ignore familial relatedness result in substantial inflation of the false-positive rate. For UNI-ADJ, only 1 variant (chr11:119059358) has a Bonferroni-corrected *p *value smaller than 0.05 (P_Bonferroni _= 0.038). It is a coding variant for gene *PDZD3*. To our knowledge, the literature has suggested no role for this gene in blood pressure. For SKAT-ADJ, there is no gene with a Bonferroni-corrected *p *value smaller than 1.

## Discussion and conclusions

In this study, we investigated the utility of linear mixed models in adjusting for familial structure. We also attempted to combine a linear mixed model method with a kernel score test--a state-of-the-art collapsing methodology that tests for association between a group of variants and a phenotype.

## Conclusions

We found that linear mixed models are able to satisfactorily adjust for familial relatedness in the sense that false-positive rates are well controlled at the nominal significance level. Not surprisingly, in terms of the statistical power, the performance of the single-variant method and the collapsing method depends on the distribution of the variants' effect sizes within the gene. The single-variant method is more powerful for genes with one dominating causal variant, whereas the collapsing method is more powerful for genes with multiple causal variants of similar effect sizes.

## Competing interests

The authors declare that they have no competing interests.

## Authors' contributions

CL and HZ designed the overall study; CY, CL, MC, XC, and LH conducted statistical analyses; and CL and HZ drafted the manuscript. All authors read and approved the final manuscript.

## References

[B1] Nielsen R Genomics: In search of rare human variants Nature 2010 467 1050 1051 10.1038/4671050a 20981085

[B2] Bansal V Libiger O Torkamani A Schork NJ Statistical analysis strategies for association studies involving rare variants Nat Rev Genet 2010 11 733 785 10.1038/nrg2825 20940738 PMC3743540

[B3] Wu MC Lee S Cai T Li Y Boehnke M Lin X Rare-variant association testing for sequence data with the sequence kernel association test Am J Hum Genet 2011 89 82 93 10.1016/j.ajhg.2011.05.029 21737059 PMC3135811

[B4] Ott J Kamatani Y Lathrop M Family-based designs for genome-wide association studies Nat Rev Genet 2011 12 465 474 21629274 10.1038/nrg2989

[B5] Kang HM Sul JH Service SK Zaitlen NA Kong SY Freimer NB Sabatti C Eskin E Variance component model to account for sample structure in genome-wide association studies Nat Genet 2010 42 348 354 10.1038/ng.548 20208533 PMC3092069

[B6] Zhou X Stephens M Genome-wide efficient mixed-model analysis for association studies 2012 44 Nat Genet 821 824 10.1038/ng.2310 PMC3386377 22706312

[B7] Wiencierz A Greven S Küchenhoff H Restricted likelihood ratio testing in linear mixed models with general error covariance structure 2011 5 Electron J Statist 1718 1734

